# Social Media in Adolescents: A Retrospective Correlational Study on Addiction

**DOI:** 10.3390/children10020278

**Published:** 2023-01-31

**Authors:** Rebecca Ciacchini, Graziella Orrù, Elisa Cucurnia, Silvia Sabbatini, Francesca Scafuto, Alessandro Lazzarelli, Mario Miccoli, Angelo Gemignani, Ciro Conversano

**Affiliations:** 1 Department of Surgical, Medical and Molecular Pathology and Critical Care Medicine, University of Pisa, 56126 Pisa, Italy; 2 Department of Civilizations and Forms of Knowledge, University of Pisa, 56126 Pisa, Italy; 3 Department of Clinical and Experimental Medicine, University of Pisa, 56126 Pisa, Italy

**Keywords:** social media addiction, anxiety, self-esteem, adolescence, developmental psychology

## Abstract

Considering the growing interest in the possible effects of internet’s addiction on adoles-cent’s mental health, this study aimed at exploring the psychological correlates of social media and internet problematic use during the first year of the covid-19 pandemic. A cross-sectional study was conducted in a sample of secondary school students (N = 258); participants were asked to complete an online survey, investigating social media addiction (BSMAS), self-esteem (RSES), feelings of isolation (CSIQ-A) and anxiety (STAI-Y). Data analysis (descriptive statistics, correlational and regression analyses) was conducted through XLSTAT software ©. An additional ad hoc questionnaire was administrated. Findings showed that the 11% of the participants were significantly addicted to social media, mostly females (59%). Gender represented an exposure factor for the hours spent on social media and the checking activity while performing other daily activities. Significant correlations emerged between the self-report measure of social media addiction and self-esteem and anxiety. Low scores at RSES corresponded to higher checking activity, hours spent on social networks, and playing videogames that were investigated as supplementary indicators of addiction with ad hoc questionnaire. The regression analysis showed just two predictors of social media addiction, gender (female) and trait anxiety. Limitations and implications of the study were argued in order to give some indications for future programs.

## 1. Introduction

During recent years and especially soon after the COVID-19 pandemic period, an increase in psychological issues and in internet/social media addiction among adults and young adults was observed [1,2,3,4] In fact, considerable rates of Internet Addiction Disorders (IAD) and Internet Gaming Disorders (IGD) are observable worldwide [5]. A demonstration of internet addiction can be found in problematic social media use, which can be addressed analyzing some risk factors such as social isolation, loneliness, low self-esteem, and anxiety, also considering the critical developmental stage of adolescence [6,7].

### 1.1. Adolescence and the Role of Sociability

Adolescence is characterized by a transitional phase from childhood to adulthood involving different physiological, cognitive, behavioral, emotional, and social changes [8]. Globally, this process occurs under social, cultural, and media influences [9,10] and it is marked with an important reorganization of logical and scientific reasoning and noticeable morphological changes which are due to the release of gonadotropins (GnRH), hormones that allow the development of primary and secondary sexual characteristics [11]. These changes occur in parallel with an intense brain reorganization known as pruning, the process that leads to the termination of some pre- and post-synaptic components to increase the plasticity and functionality of the most effective neural networks [12]. This event suggests that the “adolescent” brain may be more responsive than an “adult” brain to stressful stimuli, potentially leading to an increased vulnerability to psychopathology such as anxiety, mood, and substance abuse disorders [13]. A large part of the adolescent’s emotional well-being depends on the presence of social interactions with peers, which influence their uses and habits, favor the birth of the first sentimental relationships, and promote the construction of a private self [14]. Adolescents are particularly vulnerable to peers’ approval [15]. Social relations with peers are truly essential in promoting independence and identity realization [16]. Relationships are mainly consolidated in school contexts, where the peer group is a source of safety and a behavioral reference model [17]. However, the growth of the adolescent does not exclusively depend on social relationships; loneliness also plays an important role and assumes both physiological and pathological aspects: in particular, the latter are characterized by feelings of social isolation supported by a sense of rejection, due to the difficulty in relating to others or the feeling of exclusion [18,19].

During the time we conducted our study, or rather the COVID-19 pandemic period, perceived loneliness and social isolation were evidently highly affected by the environmental stressor of the pandemic and its restriction measures. During that time, we could expect that more adolescents needed social relationships, heading towards problematic use of the internet and social media as a compensatory conduct [20]. In future studies, it would be important to clarify whether this problematic use continued in the years to come, and what role the pandemic has played in this increase that has been observed [21,22,23,24].

In this regard, our hypothesis was to find a high prevalence of problematic social media use in adolescents (first hypothesis), and we also expected that the more they felt isolated and experienced pathological aspects of loneliness, the more they would use social media and develop a problematic use (second hypothesis). Likewise, we could assume that the opposite was true: problematic social media use may not reduce isolation but boost the feeling of loneliness.

### 1.2. The New Addictions: Internet and Social Media Misuse

Social media are internet-based applications allowing the exchange of content such as messages, thus encouraging communication with other individuals [25]. Given their massive popularity, social media have attracted considerable attention in the scientific community, with studies often pointing to controversial results. Indeed, while using social media seems to increase the circulation of ideas and information, it may also lead to negative psychological consequences [26]. In this regard, the excessive use of the internet has been an object of research since the mid-1990s, creating the definition of “addiction to technology” as a physical and behavioral dependence which shares similar features with substance addiction [27]. Subsequently, Moreno and colleagues [28] identified seven problematic internet use-related features, such as psychosocial risk, physical weakness, emotional, social, or functional weakening, dangerous use of the internet, impulsive use of the internet, and internet addiction. Despite these premises, the new manuals for statistical diagnosis do not yet recognize the use of social media and the internet as a distinct diagnostic category of disorder [29]. The existence and validity of the term “behavioral” addictions have been long discussed by the scientific community [30]. In 1980, the American Psychiatric Association (APA) workgroup on Substance Use and Related Disorders reviewed recent research and planned to identify new pathologies to be included in the manual, concluding that existing data on conditions other than Internet Gaming Disorders were too preliminary to fit inclusion criteria. At the time, internet and social media addiction may have suffered from non-standardized definitions and the applications of measures addressing unclear constructs [31].

The theoretical and motivational framework that might justify the inclusion of these new addictions draw inspiration from communication studies [32]. A useful theoretical framework adopted for the present study was the Media Dependency Theory, combined with the traditional addiction theories such as the Uses and Gratifications Theory [33]. These frames state that social media can fulfil three human social needs which are: surveillance, or the need to understand one’s social environment; social utility, or the need to act efficiently and significantly; and the need for an escape, or a way “out” when feeling overwhelmed [34]. Fulfilling these needs through social media use/abuse would create an addictive pattern [32].

Teenagers perceive social media as safe places where they feel free to express themselves [35]. However, an excessive use of social media could develop negative consequences for psychosocial life, such as a reduction in self-esteem, social isolation linked to social anxiety, and new abnormal behaviors known as FoMO and Phubbing [36,37,38]. An intense connection between anxiety and social media use was found in both males and females [39,40] in terms of increasing time spent on social media [41,42] and passive social media use [43]. In a recent study, Boursier, Gioia, and Griffiths [44], analyzing 578 selfies, found that “social appearance anxiety” (concern for other’s judgment on physical appearance) was higher in males and created a sort of vicious cycle, as it pushed them to increase their use of social media to improve their self-esteem. On the contrary, social media seemed to have a positive impact on social isolation, both directly and indirectly, by favoring a perception of increased social support [45]. In addition, according to recent studies, excessive use of social media together with depression, low self-esteem and poor social skills, tendencies towards avoidance (escaping the surrounding reality), poor school performance, and dysfunctional family environment represent the basis of internet gambling addiction [46,47].

In our sample, we expected to find higher anxiety and lower self-esteem in adolescents who use more social media and show problematic use behaviors.

Starting from these assumptions, we summarize our three hypotheses to find new answers or confirm the literature’s:

(1) Adolescents show a quite moderate prevalence of social media addiction or problematic use;
(2) Adolescents who make greater use of social media obtain higher scores on the CSIQ-A questionnaire (The Classmates Social Isolation Questionnaire for Adolescents) and generally perceive more loneliness;
(3) The problematic use of social media is positively correlated with high levels of anxiety and social isolation, and negatively correlated with self-esteem;
(4) In order to predict social media addiction, we expected anxiety, social isolation, and low self-esteem to result in risk factors.

## 2. Methods

### 2.1. Participants and Procedure

The present study follows the principles of the Declaration of Helsinki. Data were obtained from an online survey where two hundred and sixty-five adolescents (N = 265; aged 14–22 years, *M_age_* = 17.42, SD = 1.73) were asked to answer questions about their attitudes towards social media and completed validated questionnaires to observe some psychological features. Seven participants were excluded from the sample as they did not give consent for the analysis. Therefore, the final sample comprises 258 participants, of whom 58% are females (Table 1). There was no compensation of any kind for the study participants.

Data collection occurred from May to July 2020, soon after the first COVID-19-related lockdown in Italy. Participants were recruited through a convenience sampling, from a vocational school in Pisa. Students were voluntarily invited to take part in this study and provided informed consent; for underage students, the consent was provided by their parents/caregivers.

### 2.2. Measures

The first part of the online survey comprised the following questionnaires:

- Bergen Social Media Addiction Scale (BSMAS), Italian version (Cronbach’s α = 0.88; Monacis et al., 2017) [48]: it assesses the experiences in the use of social media referring to the past year. The scale uses a five-point Likert scale, ranging from 1 (very rarely) to 5 (very often). Examples of items are: You spend a lot of time thinking about social media or planning how to use it; You feel an urge to use social media more and more, etc. [49]. The BSMAS presents six items reflecting core addiction elements: salience, mood modification, tolerance, withdrawal, conflict, and relapse [27]. The scale presented a good reliability in line with previous studies (α = 0.73).
- Rosenberg Self-Esteem Scale (RSES), Italian version (Cronbach’s α = 0.84; Mannarini, 2010) [50]: it is a 10-item scale rated on a 4-point Likert scale ranging from 0 (strongly agree) to 3 (strongly disagree). It was developed by Rosenberg (1965) [51] and assesses both negative and positive feelings about the self, mainly in adolescents [52]. Examples of items are: I feel that I am a person of worth, at least on an equal plane with others; At times I think that I am no good at all (R). In the present study, the scale showed good reliability in line with the previous literature (α = 0.74).
- Classmates Social Isolation Questionnaire for Adolescents (CSIQ-A) (Cronbach’s α = 0.85; Cavicchiolo et al., 2019) [53]: it is a two-dimensional test that assesses in eight items the absence of social relationships with classmates in and out of school contexts [54]. Items should be rated on the following Likert scale: None, Few, Some, Many, All. Item examples are: How many of your classmates do you chat with?; How many of your classmates do you do activities with in your free time?). In the present study, the scale showed very good reliability (α = 0.84) with the previous literature.
- State–Trait Anxiety Inventory (STAI-Y), Italian version (Cronbach’s STAI Y1 α = 0.95; STAI Y2 α = 0.90; Pedrabissi and Santinello, 1989) [55]: designed by Spielberger and co-authors [56], it consists of 40 self-report items on a 4-point Likert scale, divided in two scales, respectively: STAI-Y1 for state anxiety and STAI-Y2 for trait anxiety. Item examples are: I feel that difficulties are piling up so that I cannot overcome them; I am presently worrying over possible misfortunes. This questionnaire was created to evaluate state and trait anxiety in adults. However, it has also been applied in adolescents [57,58]. In the present study, both scales showed very good reliability (STAI Y1 α = 0.86; STAI Y2 α = 0.87).

The second part consisted of supplementary variables, collected through an ad hoc questionnaire (see Appendix A). The investigated variables were a change in anxiety level during the social media use, perceived loneliness, perception of time flow, hours spent on social media, playing videogames, and the need to check social media while performing other activities.

## 3. Results

### 3.1. Data Analysis

The analysis was carried out on the raw scoring of the questionnaires with a significant level of 0.05. According to a Shapiro–Wilk test, the hypothesis of normality was rejected for all variables under study. Therefore, the Spearman correlation, Mann–Whitney test, and Kruskal–Wallis test with Dunn procedure were used. Lastly, the Chi-squared test or Fisher’s exact test (depending on the frequencies) were applied to identify the potential independence between categorical variables. All analyses were performed with XLSTAT version 2020 and R 4.0.3.

#### 3.1.1. Descriptive Analysis

Table 1 shows the descriptive statistics of both the total sample and social media addicts, who scored above a clinical level (≥19) on the social media addiction test.

The addicts were 11% of the total sample, which was 58% composed of females. Considering the hours spent daily using social media, three percent more than the frequency of social addicts reported spending 6 to 8 h online (14% of the total sample). There were no significant clinical scores within the sample, apart from the STAI-Y1 and STAI-Y2 results, where the average level of anxiety was labeled as “severe”.

The answers to the supplementary questions revealed that 38% of the total sample spent from 2 to 4 h daily on social media, mostly using a smartphone (91% of subjects). There was high discrepancy between the perception of anxiety using social media, for which 85% did not feel any change (“as always”) while 15% perceived a reduction in anxiety. About 70% did not feel a different perception of loneliness using social media. However, 22% of the total sample reported feeling less alone, while 10% of addicts felt lonelier using social media.

Regarding the checking of social media during other activities, most subjects (55%) declared they checked social media “from 1 to 2 times”, while 14% declared checking “every 30 min”. Moreover, 50% “rarely” or “never” played video games.

#### 3.1.2. Correlations

In the total sample (see Table 2), BSMAS was negatively correlated with RSES, and positively with STAI-Y1 and STAI-Y2, partially confirming our third hypothesis. Adolescents who were more addicted also presented low self-esteem and high state and trait anxiety. No direct correlation was found between social media addiction and social isolation.

CSIQ-A was negatively associated with trait anxiety and positively with self-esteem. Hence, the less adolescents isolated, the more they were steadily anxious, the less they had self-esteem.

Moreover, strong negative correlations were found between self-esteem and anxiety (state and trait). A weak positive correlation emerged between age and state anxiety, revealing older individuals to be more anxious.

In the subsample of addicts, the only correlations confirmed were between state and trait anxiety and the variables RSES and CSIQ-A (see Table 3). A new negative correlation appeared between state anxiety and social isolation.

#### 3.1.3. Influence of Gender

**Figure 1:**
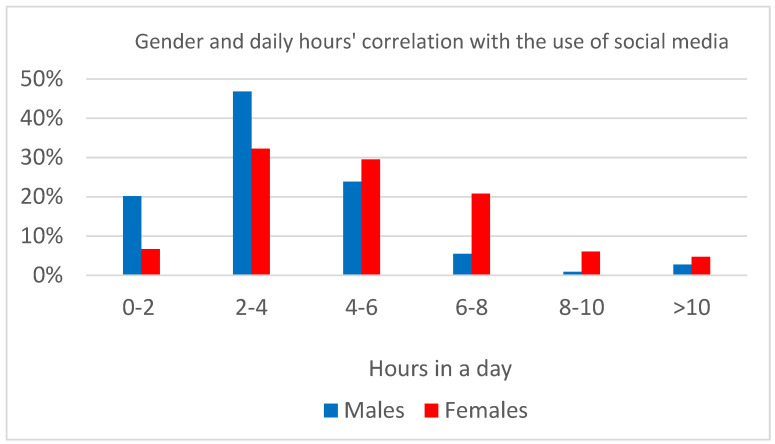
Gender differences in time spent using social media.

**Figure 2:**
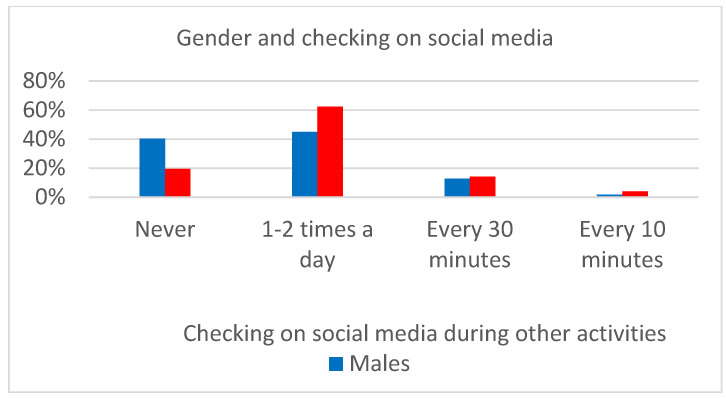
Relationship between gender and social media control tendency while carrying out other activities.

Gender differences were found in BSMAS (U = 11,227; *p* < 0.0001), self-esteem (U = 5592; *p* < 0.0001), and state (U = 10168; *p* < 0.0001) and trait anxiety (U = 11123; *p* < 0.0001). Moreover, being a woman represented an exposure factor for the hours spent on social media (*X*^2^(5) = 28.60; *p* < 0.0001, Figure 1) and checking social media while performing other activities (*X*^2^(3) = 14.26; *p* < 0.01, Figure 2).

#### 3.1.4. Regression Model

A linear regression was performed on BSMA to verify our fourth hypothesis. Sex was inserted as a dummy variable in the model. As we can observe in Table 4, only sex (female) and STAI-Y2 emerged as significant positive predictors. To be women and to score high in trait anxiety were exposure factors to developing social media addiction.

#### 3.1.5. Analysis of Supplementary Outcome Variables

**Figure 3:**
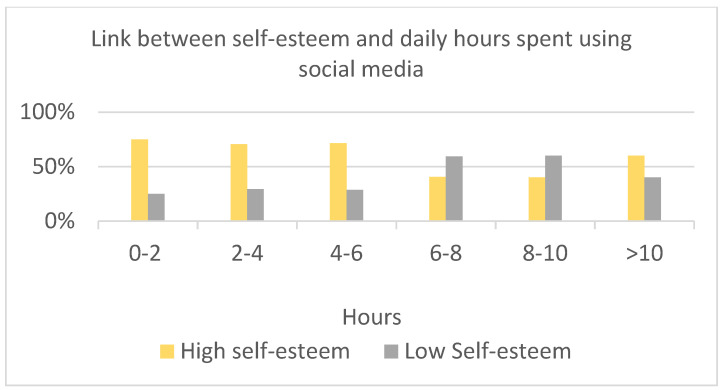
Link between self-esteem and daily hours spent using social media.

**Figure 4:**
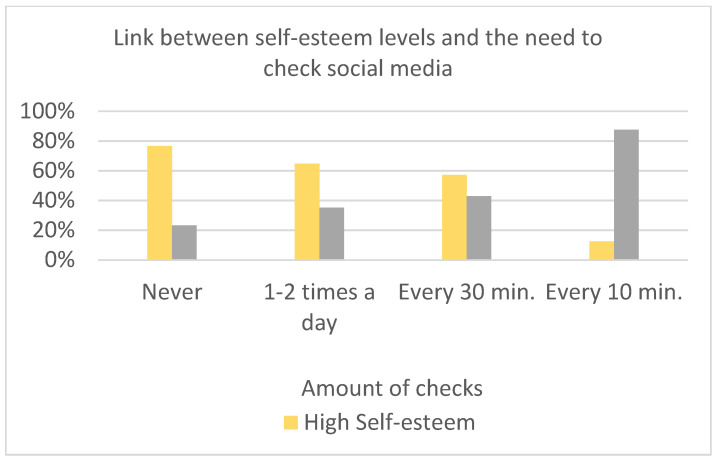
Link between self-esteem levels and the need to check social media while performing other activities.

Significant differences among means were found in hours spent using social media (K = 17.11; *p* < 0.05) (Figure 3) regarding perception of loneliness (K = 17.67; *p* < 0.001), the need to check social media (K = 10.81; *p* < 0.05) (Figure 4), and playing videogames (K = 19.18; *p* < 0.01), depending on the level of Self-Esteem (normal vs. low). Those who had low self-esteem (<15) felt lonelier and spent more time on social media, checking, and playing videogames. Feeling lonely and spending time online were positively associated (*X*^2^(3) = 23.83; *p* < 0.05).

Differences were revealed in playing videogames (K = 11.57; *p* < 0.05) regarding the level of state anxiety and in the perception of time flow (K = 6.25; *p* < 0.05), depending on trait anxiety. Anxious adolescents played more videogames and perceived faster time flow.

## 4. Discussion

The aim of the present study was to investigate the increasingly prevalent problematic behavior related to adolescents’ social media use in light of the emergency COVID-19 pandemic period [41,59]. We hypothesized that there was a widespread problematic use of social media in adolescence which increased during pandemic, was positively correlated with anxiety, and was negatively correlated with self-esteem. We also expected that adolescents who made greater use of social media showed higher social isolation and perceived generally more loneliness.

(1) Adolescents show a fairly moderate prevalence of social media addiction or problematic use.
(2) Adolescents who make greater use of social media obtain higher scores on the CSIQ-A questionnaire (The Classmates Social Isolation Questionnaire for Adolescents) and generally perceive more loneliness.
(3) The problematic use of social media is positively correlated with high levels of anxiety, and negatively correlated with self-esteem.
(4) In order to predict social media addiction, we expected anxiety, social isolation, and low self-esteem to be risk factors

### 4.1. Social Media Addiction Prevalence

Approximately 11% of the sample was found to be addicted to social media, which was consistent with numerous previous studies [60,61,62] and with our first hypothesis. This percentage increased up to 14% when we considered subjects who spent 6 to 8 h on social networks daily.

### 4.2. Social Media Addiction and Gender

Regarding gender differences, we found most addicts were women. An explanation could be that women were driven to maintain these social media attitudes due to lower self-esteem compared to men [63]; indeed, in our sample, they showed lower self-esteem, in accordance with previous results [64], and higher anxiety. Other studies, for instance, revealed gender differences for depressive reactions to trauma, showing a higher vulnerability to develop DSM-5 PTSD in women in comparison to men [65].

### 4.3. Social Media Addiction and Psychological Variables

BSMA was positively correlated to all the other psychological variables but isolation, partially confirming our hypotheses.

The association between addiction, anxiety, and low self-esteem is in line with previous results [42,44].

Looking at the supplementary variables, we found a significant negative link between self-esteem on one side and hours spent on social networks, checking social media, and playing videogames on the other side. Checking online posts distracts from other activities, which is representative of failed self-control, fueling the use of social media [66]. This, to the knowledge of the authors, is the first study where a significant link between self-control behavior, such as checking social media, and self-esteem appears.

We did not find a correlation between addiction score and social isolation, but we found that hours spent using social media were positively correlated with perceived loneliness, in accordance with Twenge and colleagues [67], who found intense social media users experienced a greater sense of loneliness.

Counterintuitive results emerged regarding social isolation that was positively associated with self-esteem and negatively with anxiety. The more adolescents had higher self-esteem, the more they were socially isolated with the rest of the class. To interpret this result, we should premise that class social isolation was also an expected result of online lessons and restrictive measures during the COVID-19 pandemic. Hence, adolescents who were deprived of in-person peer socialization could be more prone to developing higher level of self-esteem as a defensive response to the lack of social recognition in real interactions. Indeed, the valuation of self-image, underling the construct of self-esteem, does not necessarily correspond to a recognition of a true self, and to an authentic self-awareness, more underlined in the construct of self-compassion [68].

Regarding the negative correlation with anxiety, the result could be also interpreted considering the fear of social interaction during Covid, thus the more adolescents were isolated in the real life, the less they were anxious. The norm of social restriction might have enhanced social anxiety and, consequently, increased misuse of social media to meet the natural need of social engagement.

Therefore, people with higher anxiety, had lower self-esteem, were more likely to play videogames and perceive faster time’s flow. However, most subjects presented severe levels of state and trait anxiety, thus indicating a baseline anxiety, and their perceived anxiety did not change during the use of social networks. This result could support the thesis of a tendency among adolescents to experience anxiety regardless of social media use [42,69].

Considering the regression model, beyond gender, anxiety was the only direct predictor of social media addiction. It was intriguing to observe that trait and not state anxiety was a significant predictor, along with the literature that identified a more durable disposition of anxiety to predict a dysfunctional addictive behavior [43,70].

### 4.4. Limitations

This study presents some methodological limitations. Firstly, the self-report measures included explicit questions that do not exclude the problem of social desirability. To measure social media addiction, for instance, we should consider that social media addicts might have misperception about their risk behaviors, and about the speed of time flow [71]. Hence, we suggest the use of behavioral measures regarding daily routine to assess social media addiction [72] and reduce the problem of underestimation of problematic behaviors. A qualitative approach, such as a daily diary method, could be combined. Second, we only evaluated isolation among adolescents with classmates during online teaching. This does not exclude the possibility that an individual can perceive isolation within the class and, at the same time, have good interpersonal relationships outside of class and vice versa, especially during school restrictions on in-person interactions.

## 5. Conclusions

Given these results, some indications for future interventions can be highlighted. For instance, interventions aiming to implement more authentic self-awareness, rather than feeding an external self-image, and at the same time improving attention, emotional regulation, and reducing anxiety, might be encouraged [73,74]. Interventions that could help adolescents in increasing their psychophysical well-being concern mindfulness-based interventions and cognitive behavioral psychoeducational programs [75,76,77]. Mindfulness interventions were shown to be effective in addiction and physical [78,79], and emotional and cognitive problems in adolescents [80].

In addition, the prevalence of females in the problematic use of social media should be explored in subsequent studies to fully understand the predominantly gender-related characteristics that make females more vulnerable to the use of social media.

Further studies are necessary to understand what the risk and resilience factors are in adolescence, to prevent addiction and distinguish it from a regular social media use.

As result of our study, feeling more isolated during the pandemic was paradoxically a protective factor from developing addiction. To bear a sense of solitude might have prevented adolescents from excessive social media use which could foster addiction.

Moreover, further factors that could play a key role in the prevention of social media addiction should be studied, such as mindfulness and relatedness to nature, since both promote restorative attention and well-being [81]. Programs that enhance relatedness to nature in outdoor activities, together with attention to the present moment, could promote a more balanced use of tech by promoting psychological well-being as well. Finally, research should deepen the understanding and the intervention on what is missed in our community contexts, including schools, which encourage the misuse of social media. A current challenge is to build in-person social relationships that reinforce self-esteem, emotional regulation, and the use of functional coping strategies to deal with global environmental stressors. Addiction could be considered a dysfunctional emotion-oriented strategy to cope with stable anxiety and stressful events, increased above all in the time of environmental stressors such as the pandemic [82]. Stressors such as the pandemic recall, for instance, the fear of death and, thus, require new skills in practitioners [83] and training programs that consider the psycho-immunological interconnection [84] to reinforce resilience and self-care, even in time of crisis.

## Figures and Tables

**Table 1:** Descriptive statistics in the total sample (N = 258) and social media addicts sample (N = 29). M = Males; F = Females.

	Total Sample		Social Media Addicts	
Variables	Frequencies (%)	*M* (SD)	Frequencies (%)	*M* (SD)
Sex	M = 42.25		M = 41.38	
	F = 57.75		F = 58.62	
Age		17.42 (1.73)		17.86 (1.48)
School failure	No = 64.73		No = 75.86	
	Yes = 35.27		Yes = 24.14	
BSMAS		13.07 (4.48)		21.76 (2.46)
RSES		16.59 (5)		16.17 (4.90)
CSIQ-A		21.35 (5.14)		19.45 (4.95)
STAI-Y1		42.67 (9.93)		46.86 (10.67)
STAI-Y2		45.88 (10.22)		52.52 (11.50)

**Table 2:** Spearman correlations in the total sample (N = 258).

	Age	BSMAS	RSES	CSIQ-A	STAI-Y1	STAI-Y2
Age		0.02	0.01	−0.07	0.24 **	0.12
BSMAS			−0.30 **	−0.06	0.30 **	0.41 **
RSES				0.26 **	−0.51 **	−0.75 **
CSIQ-A					−0.10	−0.20 **
STAI-Y1						0.72 **
STAI-Y2						

** *p*-value < 0.01.

**Table 3:** Spearman correlations in social media addicts (N = 29).

	Age	BSMAS	RSES	CSIQ-A	STAI-Y1	STAI-Y2
Age		0.09	0.18	−0.09	0.18	−0.11
BSMAS			0.12	0.22	−0.05	0.13
RSES				0.31	−0.44 *	−0.81 **
CSIQ-A					−0.38 *	−0.37 *
STAI-Y1						0.43 *
STAI-Y2						

* *p*-value < 0.05; ** *p*-value < 0.01.

**Table 4:** Linear regression on BSMA.

Variables	B	SE	t	*p*-Value	Lower (95%) CI	Upper 95% CI
Constant	2.84	2.84	0.99	0.32	−5.04	6.04
Sex (Female)	2.15	0.53	4.03	0.00	1.10	3.20
RSES	0.07	0.07	0.98	0.32	−0.07	0.21
CSIQ-A	0.005	0.05	0.10	0.92	−0.09	0.10
STAI-Y1	−0.03	0.03	−0.92	0.35	−0.10	0.04
STAI-Y2	0.20	0.04	4.45	0.00	0.11	0.28

R^2^ = 0.23; F = 14.80, *p* < 0.01.

## Data Availability

The data that support the findings of this study are openly available in Mendeley Data at: https://data.mendeley.com/datasets/vftw9cz723/1 (second revision version accessed on 31 January 2023), DOI: 10.17632/vftw9cz723.1
